# Anterior placenta previa in the mid-trimester of pregnancy as a risk factor for neonatal respiratory distress syndrome

**DOI:** 10.1371/journal.pone.0207061

**Published:** 2018-11-02

**Authors:** Ki Hoon Ahn, Eun Hee Lee, Geum Joon Cho, Soon-Cheol Hong, Min-Jeong Oh, Hai-Joong Kim

**Affiliations:** 1 Department of Obstetrics and Gynecology, Korea University College of Medicine, Seoul, South Korea; 2 Department of Pediatrics, Korea University College of Medicine, Seoul, South Korea; Azienda Ospedaliera Universitaria Policlinico di Bari, Universita' degli Studi di Bari, ITALY

## Abstract

This study investigated whether anterior placenta previa in the second trimester is associated with neonatal respiratory distress syndrome (RDS). The neonates delivered by 2067 women between 2007 and 2015 were evaluated for the presence of RDS through birth records. The location of the placenta and the presence of placenta previa during the second and third trimesters were assessed and recorded. Demographic, prenatal, and perinatal records were reviewed. Anterior placenta previa in the second and third trimesters was correlated with RDS. Infants with lower gestational ages and birth weights had higher rates of RDS. Multivariate logistic regression analysis identified a significant association between anterior placenta previa in the second trimester and neonatal RDS. Anterior placenta previa in the second trimester is associated with neonatal RDS. Obstetricians should be aware that anterior placenta previa detected during the second trimester, irrespective of whether the placenta will migrate in the third trimester, may be an independent risk factor for neonatal RDS.

## Introduction

Respiratory distress syndrome (RDS) in neonates reflects the immature condition of the fetal lung. The incidence of RDS is inversely correlated with the gestational age and birth weight of the infant [1]. Known risk factors for RDS are maternal diabetes, cesarean delivery, male sex of the infant, neonatal sepsis, asphyxia, and prematurity [2–5].

Women with placenta previa are generally more prone to deliver babies with RDS [6,7]; however, data on the association between placenta previa and neonatal RDS have been inconsistent [8]. Because cesarean delivery is commonly performed in women with placenta previa to prevent uncontrolled bleeding irrespective of fetal lung maturation, prematurity of the neonates might be a major contributor to these inconsistent data.

In addition, the position of placentation has been suggested to affect pregnancy outcomes. Particularly, anterior placentation, compared with posterior placentation, is more highly associated with adverse perinatal outcomes, such as fetal growth restriction and preterm births, as well as higher rates of premature rupture of membranes (PROM) and cesarean section, which are known risk factors for neonatal RDS [9,10].

Based on the relationship between anterior placentation and adverse perinatal outcomes, anterior placenta previa can be suspected as a risk factor for neonatal RDS. However, no report has evaluated the correlation between the specific location of placenta previa and neonatal RDS.

Therefore, this study aimed to investigate whether a greater association is found between anteriorly located placenta previa (hereafter, anterior placenta previa) and neonatal RDS than that between posteriorly located placenta previa (hereafter, posterior placenta previa) and RDS. The relationship between neonatal RDS and the location of placenta previa based on the gestational trimester was also evaluated.

## Materials and methods

The institutional review board of the university hospital approved this case–control study (AN15039-003) and waived the requirement for informed consent. Data were retrospectively collected by reviewing the medical records of 2770 women who delivered between March 1, 2007, and March 31, 2015. All data of live births were included. Exclusion criteria include insufficient ultrasonographic data, multiple pregnancies, and absence of neonatal data. Neonatal RDS was defined as respiratory difficulty caused by surfactant deficiency [11]. Data on this condition were collected from pediatric charts. Women whose neonates do not have RDS served as the control group. Eventually, 2067 women were included in the analyses.

Basic demographic characteristics or possible confounding factors included age (in years) at delivery, height (cm), weight (kg), body mass index (BMI, kg/m^2^), and parity. Blood glucose levels (mg/dL) were recorded using a 50-g oral glucose tolerance test. We evaluated the following delivery factors related to neonatal health: sex of the infant, birth weight, and gestational age at delivery. In addition, maternal diseases, including hypertension (including chronic and pregnancy-associated hypertension), diabetes mellitus (including pregestational and gestational diabetes), and thyroid dysfunction (including overt and subclinical hyper- and hypothyroidism), were investigated. Indications for cesarean delivery include the following: previous cesarean delivery; dystocia; fetal distress based on electronic heart rate tracings; abnormal presentation, such as breech and transverse lie; placenta previa; fetal growth restriction; oligohydramnios; multiple pregnancies; large-for-gestational-age fetuses; uncontrolled preeclampsia; maternal diseases, such as genital infections, pelvic bone abnormalities, epilepsy, and uterine myoma; genital conditions suggestive of labor obstruction; pelvic mass requiring combined surgery; fetal anomalies; maternal request; and others (previous uterine surgeries, severe vulvar vascular congestion, and older primigravida).

At our hospital, trained staff members routinely check for placenta previa via transvaginal ultrasonography between 19 and 24 weeks of gestation in the second trimester and at 37 weeks of gestation in the third trimester. Routine ultrasonography before delivery is performed at 37 weeks of gestation in the third trimester. Placenta previa is observed every 2 or 3 weeks until delivery in cases where placenta previa was detected ultrasonographically in the second trimester. In the present study, to define placental location, an ultrasound probe was applied perpendicularly to the abdominal wall of a pregnant woman, at the center of the uterus. The placental location was longitudinally assessed with respect to the uterine axis and recorded. Based on this assessment, the placental site was determined to be either the anterior or the posterior uterus. Gestational age via ultrasonography, fetal body weight percentile auto-calculated using an ultrasound machine [12], and the single deepest pocket estimation of amniotic fluid volume were recorded. In total, 98% of the study participants were Koreans who could speak Korean. Therefore, neither ethnic nor racial differences were factored into our analyses.

An antenatal corticosteroid was administered for fetal lung maturation if preterm birth was expected between 24 and 34 weeks of gestation. Trained staff collected all data.

Continuous variables were expressed as mean±standard deviation in normally distributed cases; alternatively, the median (range) was used. Continuous variables were compared between the RDS and non-RDS groups using the Student *t*-test or Mann–Whitney *U* test, depending on the distribution of the variables. The differences in percentages between the groups were analyzed using Pearson Chi-square or Fisher exact test. Multivariate logistic regression analyses were performed for neonatal RDS. Odds ratios (ORs) and 95% confidence intervals (CIs) were calculated to determine the factors associated with RDS in neonates. All statistical analyses were performed using SPSS version 19.0 (SPSS, Inc., Chicago, IL, USA). Two-sided tests of significance were used, and a P value of <0.05 was considered statistically significant for all analyses. A post-hoc sample size power calculation indicated that the inclusion of 2067 participants was sufficient to attain a statistical power of 80% and an alpha error of 5%.

All relevant data are within the manuscript.

In this article, the clinical definition of terminologies is as follows.

Respiratory distress syndrome: respiratory distress requiring ventilator care with surfactant therapy

Placenta previa: total, partial, and marginal coverage of the placenta on the internal os of the cervix

Maternal hypertension: chronic hypertension and pregnancy-associated hypertension, including gestational hypertension, preeclampsia, and eclampsia

Diabetes mellitus: pregestational and gestational diabetes

Hyperthyroidism: a suppressed serum thyroid-stimulating hormone value and a high free T4 and/or free T3 level in our institute laboratory reference for pregnancy

Hypothyroidism: a high serum thyroid-stimulating hormone value and a low free T4 and/or free T3 level in our institute laboratory reference for pregnancy

## Results

In total, 78 and 1989 women in the RDS and non-RDS groups, respectively, were included in the analyses. No differences were found in the maternal demographic characteristics between women in the neonatal RDS and non-RDS groups, including age, BMI, and medical and surgical histories. Analysis of perinatal data showed that neonates with lower gestational ages and body weights at birth had a higher risk of neonatal RDS (P<0.001 for both) (Table 1).

**Table 1:** Maternal demographic, prenatal, and perinatal characteristics.

	RDS (n = 78)	Non-RDS (n = 1989)	P value
**Age (years)**	32.9±4.3	32.2±4.5	0.191
**Height (cm)**	160.0±5.4	160.4±5.3	0.491
**Weight (kg)**	65.4±12.5	66.8±12.9	0.332
**Body mass index (kg/m**^**2**^**)**	25.5±4.5	26.0±4.8	0.372
**Parity**	0.6±0.7	0.7±0.8	0.406
**Hypertension, n (%)**	6 (7.7)	99 (5.0)	0.29
**Diabetes mellitus, n (%)**	17 (22.2)	276 (13.9)	0.116
**Thyroid dysfunction, n (%)**			0.058
** Hyperthyroidism**	4 (5.1)	30 (1.5)	
** Hypothyroidism**	3 (3.8)	66 (3.3)	
**Prior cesarean delivery, n (%)**	21 (26.9)	467 (23.5)	0.497
**Prior conization of the uterine cervix, n (%)**	2 (2.6)	16 (0.8)	0.143
**History of pelvic inflammatory disease, n (%)**	1 (1.3)	20 (1.0)	0.424
**Serum glucose**^†^ **(mg/dL)**	124.2±31.1	117.0±29.0	0.092
**Second trimester ultrasonography**			
** Gestational age (weeks)**	22.8±3.5	22.2±3.4	0.177
** Fetal body weight (kg)**	0.62±0.4	0.6±0.4	0.323
** Fetal presentation**			
** Breech presentation, n (%)**	16 (20.0)	545 (27.4)	0.365
** Transverse lie, n (%)**	6 (7.5)	88 (4.4)	
**Ultrasonography just before delivery**			
** Gestational age (weeks)**	28.7±2.2	37.4±9.7	**<0**.001*
** Fetal body weight (kg)**	1.2±0.3	3.0±0.5	**<0**.001*
** Fetal presentation**			0.443
** Breech presentation, n (%)**	11 (14.3)	95 (4.8)	
** Transverse lie, n (%)**	7 (8.6)	8 (0.4)	
**Gestational age at delivery (weeks)**	30.9±4.1	38.4±2.0	**<0**.001*
**Preterm birth <37 weeks, n (%)**	67 (85.9)	219 (11.7)	**<0**.001*
**Use of antenatal corticosteroid, n (%)**	6 (7.1)	41 (2.1)	**0.007**
**Fetal body weight at delivery (kg)**	1.6±0.8	3.2±0.5	**<0**.001*
**Small-for-gestational age <10th percentile, n (%)**	15 (19.1)	229 (11.5)	0.058
**Low birth weight**^‡^**, n (%)**	61 (78.2)	305 (15.3)	**<0**.001*
**Female sex of infant, n (%)**	39 (49.4)	963 (48.4)	0.908
**Cesarean delivery, n (%)**	68 (87.5)	1002 (50.4)	**<0**.001*
***Indications for cesarean delivery***			
** Placenta previa**	10 (15.0)	34 (3.4)	**<0**.001*
** Previous cesarean section**	17 (25.0)	352 (35.0)	**0.037**
** Dystocia**	4 (5.9)	232 (23.2)	0.38
** Abnormal presentation**	4 (5.9)	91 (9.1)	0.307
** Fetal distress**	20 (30.0)	88 (8.8)	**<0**.001*
** Fetal growth restriction**	0 (0)	10 (1.0)	0.86
** Oligohydramnios**	0 (0)	24 (2.4)	0.788
** Multiple pregnancy**	0 (0)	13 (1.3)	0.839
** Large-for-gestational age**	0 (0)	20 (2.0)	0.803
** Preeclampsia**	7 (10.0)	17 (1.7)	**<0**.001*
** Maternal disease**	3 (5.0)	57 (5.7)	0.203
** Fetal anomaly**	0 (0)	17 (1.7)	0.82
** Maternal request**	0 (0)	3 (0.3)	0.919
**Others**^δ^	3 (5.0)	44 (4.4)	0.171

RDS, respiratory distress syndrome; NA, not applicable.

Data are presented as mean±standard deviation.

^†^In the 50-g oral glucose tolerance test.

^‡^Birth weight <2500 g.

^δ^Included previous uterine surgeries, severe vulvar vascular congestion, and older primigravida.

*Statistically significant.

The rate of preterm birth in the RDS group was higher (P<0.001) than that in the non-RDS group. Antenatal corticosteroid for fetal lung maturation in the RDS group was more frequently used than that in the non-RDS group (P = 0.007).

The frequency of placenta previa in the second trimester was significantly higher than that in the third trimester: 4.2% (86/2067) vs. 2.2% (45/2067) (Table 2).

**Table 2:** Relationship between placenta previa in the second trimester and time just before delivery and neonatal respiratory distress syndrome.

	RDS (n = 78)	Non-RDS (n = 1989)	P-value
**Second trimester**			
** No placenta previa, n (%)**	64 (82.0)	1,917 (96.4)	0.004*
** Placenta previa, n (%)**	14 (18.0)	72 (3.6)	
**Time just before delivery**			
** No placenta previa, n (%)**	67 (85.7)	1,955 (98.3)	<0.001*
** Placenta previa, n (%)**	11 (14.3)	34 (1.7)	

RDS, respiratory distress syndrome.

*Statistically significant.

Moreover, pregnancies with placenta previa were associated with a higher incidence of neonatal RDS than were pregnancies without placenta previa in the second and third trimesters (P = 0.004 and <0.001, respectively; Table 2).

When the association between placenta previa and neonatal RDS was analyzed with respect to the uterine location of the placenta previa in the second and third trimesters, only anterior placenta previa (P = 0.003 and 0.009, respectively) was significantly associated with neonatal RDS (Table 3).

**Table 3:** Relationship between the location of placenta previa and neonatal respiratory distress syndrome.

	RDS (n = 78)	Non-RDS (n = 1989)	P-value
**Second trimester**			0.001*
** No placenta previa, n (%)**	64 (82.0)	1,917 (96.4)	
** Anterior placenta previa, n (%)**	7 (9.0)	11 (0.5)	0.003*
** Posterior placenta previa, n (%)**	7 (9.0)	61 (3.1)	0.087
**Time just before delivery**			<0.001*
** No placenta previa, n (%)**	69 (88.5)	1,955 (98.3)	
** Anterior placenta previa, n (%)**	3 (3.8)	8 (0.4)	0.009*
** Posterior placenta previa, n (%)**	6 (7.7)	26 (1.3)	0.443

RDS, respiratory distress syndrome.

*Statistically significant.

No significant association between posterior placenta previa and neonatal RDS was found.

After adjusting for confounding variables including the gestational age at birth, birth weight, use of antenatal corticosteroid, and cesarean delivery, multivariate logistic regression analysis for neonatal RDS showed that the gestational age at birth and anterior placenta previa in the second trimester remained significant factors (odds ratio [OR], 0.4; 95% confidence interval [CI], 0.3–0.6; P < 0.001 and OR, 4.6; 95% CI, 2.6–25.4; P = 0.006, respectively). However, anterior placenta previa in the third trimester was not a significant factor (Table 4).

**Table 4:** Multivariate logistic regression analysis of anterior placenta previa for neonatal respiratory distress syndrome^†^.

Neonatal respiratory distress syndrome	OR^‡^	95% CI^‡^	P-value^‡^	OR^§^	95% CI^§^	P-value^§^
**Gestational age at birth**	0.4	0.3–0.6	<0.001*	0.6	0.2–3.3	0.456
**Birth weight of infant**	1.2	0.27–5.4	0.81	4.7	0.2–8.7	0.324
**Use of antenatal corticosteroid**	1.2	0.1–12.3	0.474	1.2	0.6–2.4	0.124
**Cesarean delivery**	2.7	0.3–28.7	0.41	4.6	0.9–24.7	0.07
**Anterior placenta previa**	4.6	2.6–25.4	0.006*	4.9	0.3–18.4	0.652

OR, odds ratio; CI, confidence interval.

^†^The analysis was adjusted for gestational age at birth, birth weight, antenatal corticosteroid, cesarean delivery, and anterior placenta previa.

^‡^The analysis was performed with data only for anterior placenta previa in the second trimester.

^§^The analysis was performed with data only for anterior placenta previa just before delivery.

*Statistically significant.

## Discussion

The most notable finding in our study was that the presence of placenta previa in the second trimester is associated with higher rates of neonatal RDS, and that the overall frequency of placenta previa is lower in the third trimester. Our findings are consistent with previous reports of low placentation in the second trimester leading to postpartum hemorrhage, even if the low placental position disappeared in the third trimester [13–15]. Both anterior placentation and placenta previa have been reported to be individual factors that can negatively affect neonatal respiratory outcomes [7,16]. In a study on pregnancy outcomes at term by Schneiderman and Balayla, the rate of neonatal respiratory distress due to the presence of placenta previa was higher than that due to the absence of placenta previa in the group of patients who underwent cesarean delivery [7]. Therefore, placenta previa might independently and adversely affect neonatal respiratory function.

A previous study investigating the effect of placenta previa on neonatal respiration showed that the cortisol level in the cord blood in the placenta previa group was lower than that in the control group, suggesting that lower cortisol level might contribute to the deterioration of neonatal respiratory function [6]. In addition, the amniotic fluid lamellar body count (LBC), a predictor of fetal lung maturity, has been recently reported to be lower in women with placenta previa. Lower amniotic fluid LBC was also associated with neonatal transient tachypnea, which further supports our findings [16].

If placenta previa negatively affects neonatal respiratory function, it is important to address why women with anterior placentation had an even higher rate of neonatal RDS in our study. Hence, the effects of anterior placenta previa need to be evaluated with respect to the specific pregnancy period, particularly the ante- and intrapartum periods. In the intrapartum period, anterior placentation, compared with posterior placentation, could result in a greater extent of hemorrhage [17]. The large amount of bleeding caused by anterior placentation could, in turn, lead to a higher rate of neonatal anemia, which is closely linked to neonatal RDS [6,18–20].

Meanwhile, during the antepartum period, anterior placentation might affect the fetal lung in a manner different from that in which it affects other sites of placentation; however, to the best of our knowledge, no studies have yet investigated the differences between anterior and posterior placentation with respect to fetal lung maturation. Therefore, to address the indirect effects of the location of the placenta on neonatal RDS, we examined the physiological differences between anterior and posterior placentation. Clinically, anterior placentation is associated with adverse pregnancy outcomes, including fetal growth restriction and preterm birth [9]. Based on our data, although preterm birth was the most significant risk factor for neonatal RDS, anterior placenta previa also seems to independently affect neonatal respiratory dysfunction. In addition, anterior placentation may be related to dysfunctional labor, including a prolonged third stage, higher labor induction rate, and greater incidence of manual placenta removal [10].

Durnwald and Mercer calculated myometrial thickness based on the uterine site, which helped elucidate the physiological differences between anterior and posterior placentation. The authors evaluated the myometrial thickness during all three trimesters of pregnancy and reported that the myometrial wall was thinner at the site of anterior placentation than at other sites [21]. In addition, the anterior myometrial wall tended to be thinner than the posterior and fundal myometrial walls in all pregnancy trimesters. Based on these findings and on the potential association between anterior placentation and fetal growth restriction [10], it can be concluded that the anteriorly implanted placenta and the surrounding uterine wall might be poorly vascularized compared with other placentation sites. However, in one study that analyzed uteroplacental blood flow and placental location, no statistically significant differences between the systolic/diastolic ratios of uteroplacental vessels in the anterior and posterior placentation were reported [22]. Thus, future large-scale studies thoroughly examining the relationship between uteroplacental blood flow and placental location are necessary to validate these findings.

Consistent with the results of previous studies, we identified a relatively high incidence of placenta previa during the second trimester that spontaneously resolved during the third trimester [23]. Although posterior placenta previa was generally more prevalent in the second trimester, anterior placenta previa was primarily associated with neonatal RDS during this trimester. Interestingly, anterior placenta previa during the third trimester was not associated with neonatal RDS. A trial of vaginal labor is frequently permitted when placenta previa spontaneously resolves at term, enabling physicians to overlook the potential harm that could result from this condition. Women with anterior placenta previa during the second trimester should be counseled regarding the likelihood of neonatal RDS, even if placenta previa disappears just before delivery. Our results are consistent with those of other studies, which showed that resolved placenta previa persists as a risk factor for hemorrhage at delivery [22,23].

Despite the novelty of our findings, some limitations need to be addressed. First, our study did not include factors related to cesarean section, including bleeding and transfusion volumes, operative duration, and placental pathologies related to inflammation and villous development. These factors should be included in further studies because maternal hemorrhage, cesarean section, neonatal anemia, and neonatal hypoxia secondary to repeated antepartum bleeding have been proposed as potential causes of neonatal RDS in the context of placenta previa [7,8,18].

Second, a rather small group of neonates delivered preterm was compared with a much larger group delivered at term. Because of the relatively small sample size of the RDS group, reaching a definitive conclusion concerning the link between placental location and neonatal RDS was difficult. However, the inclusion of all consecutive patients would compensate for the potential biases from a small number of participants. Third, although trained staff performed ultrasonography, verification of inter-sonographer variability was impossible because of the retrospective nature of this study. Finally, other complication outcomes were not considered as variables in our analyses because the topic of this study was neonatal RDS.

Despite its limitations, we believe that our study may have important clinical implications. When placenta previa is suggested based on mid-trimester ultrasonography findings, physicians should specify the location of the placenta (anterior or posterior), preferably with transvaginal ultrasonography. Considering the potential relationship with neonatal RDS, women who are diagnosed with anterior placenta previa using mid-trimester ultrasonography might be better cared for in a tertiary hospital, where appropriate neonatal care is available during delivery.

## Conclusion

Anterior placenta previa detected during the second trimester, irrespective of whether the placenta migrates in the third trimester, may be an independent risk factor for neonatal RDS. Our data might be useful during obstetric counselling regarding pregnancy outcomes related to neonatal RDS.
